# In-depth Profiling of MvfR-Regulated Small Molecules in *Pseudomonas aeruginosa* after Quorum Sensing Inhibitor Treatment

**DOI:** 10.3389/fmicb.2017.00924

**Published:** 2017-05-24

**Authors:** Giuseppe Allegretta, Christine K. Maurer, Jens Eberhard, Damien Maura, Rolf W. Hartmann, Laurence Rahme, Martin Empting

**Affiliations:** ^1^Department of Drug Design and Optimization, Helmholtz Institute for Pharmaceutical Research SaarlandSaarbrücken, Germany; ^2^Department of Surgery and Department of Microbiology and Immunobiology, Harvard Medical School, BostonMA, United States; ^3^Department of Surgery, Center for Surgery, Innovation and Bioengineering, Massachusetts General Hospital, BostonMA, United States; ^4^Shriners Hospitals for Children, BostonMA, United States; ^5^Pharmaceutical and Medicinal Chemistry, Saarland UniversitySaarbrücken, Germany

**Keywords:** *Pseudomonas aeruginosa*, quinolones, 2′-aminoacetophenone, dihydroxyquinoline, persistence, Quorum Sensing Inhibitors, MvfR

## Abstract

*Pseudomonas aeruginosa* is a Gram-negative bacterium, which causes opportunistic infections in immuno-compromised individuals. Due to its multiple resistances toward antibiotics, the development of new drugs is required. Interfering with Quorum Sensing (QS), a cell-to-cell communication system, has shown to be highly efficient in reducing *P. aeruginosa* pathogenicity. One of its QS systems employs *Pseudomonas* Quinolone Signal (PQS) and 4-hydroxy-2-heptylquinoline (HHQ) as signal molecules. Both activate the transcriptional regulator MvfR (Multiple Virulence Factor Regulator), also called PqsR, driving the production of QS molecules as well as toxins and biofilm formation. The aim of this work was to elucidate the effects of QS inhibitors (QSIs), such as MvfR antagonists and PqsBC inhibitors, on the biosynthesis of the MvfR-regulated small molecules 2′-aminoacetophenone (2-AA), dihydroxyquinoline (DHQ), HHQ, PQS, and 4-hydroxy-2-heptylquinoline-*N*-oxide (HQNO). The employed synthetic MvfR antagonist fully inhibited *pqs* small molecule formation showing expected sigmoidal dose-response curves for 2-AA, HQNO, HHQ and PQS. Surprisingly, DHQ levels were enhanced at lower antagonist concentrations followed by a full suppression at higher QSI amounts. This particular bi-phasic profile hinted at the accumulation of a biosynthetic intermediate resulting in the observed overproduction of the shunt product DHQ. Additionally, investigations on PqsBC inhibitors showed a reduction of MvfR natural ligands, while increased 2-AA, DHQ and HQNO levels compared to the untreated cells were detected. Moreover, PqsBC inhibitors did not show any significant effect in PA14 *pqsC* mutant demonstrating their target selectivity. As 2-AA is important for antibacterial tolerance, the QSIs were evaluated in their capability to attenuate persistence. Indeed, persister cells were reduced along with 2-AA inhibition resulting from MvfR antagonism, but not from PqsBC inhibition. In conclusion, antagonizing MvfR using a dosage capable of fully suppressing this QS system will lead to a favorable therapeutic outcome as DHQ overproduction is avoided and bacterial persistence is reduced.

## Introduction

*Pseudomonas aeruginosa* is a ubiquitous Gram-negative bacterium able to cause severe chronic infections in immuno-compromised patients, for example in people affected by cystic fibrosis (Gómez and Prince, 2007) or thermally injured individuals (Tredget et al., 2004). The eradication of this pathogen with antibiotic treatments is becoming more and more difficult because of its intrinsic and acquired resistance (Hancock and Speert, 2000; Aloush et al., 2006) and tolerance (Mulcahy et al., 2010) toward these drugs. A new promising strategy for treating *P. aeruginosa* infections is blocking its pathogenicity without killing the bacteria targeting a cell-to-cell communication system called Quorum Sensing (QS) (Hurley et al., 2012).

This bacterium employs four QS systems interconnected to each other, namely *las*, *iqs*, *pqs*, and *rhl*, for regulating the expression of several toxins needed for adjusting its metabolism and virulence during the course of infection (Lee and Zhang, 2015). The *pqs* QS system is selectively expressed by *P. aeruginosa* and utilizes the signal molecule *Pseudomonas* Quinolone Signal (PQS) and its precursor 4-hydroxy-2-heptylquinoline (HHQ) for activating the transcriptional regulator MvfR (Multiple Virulence Factor Regulator), also called PqsR. This protein induces the production of different toxins, such as lectins, pyocyanin, and hydrogen cyanide. It also regulates the expression of enzymes needed for the biosynthesis of its natural ligands encoded by the *pqsABCDE* operon (Xiao et al., 2006) and has been shown to be essential for persister cells development (Que et al., 2013).

**FIGURE 1:**
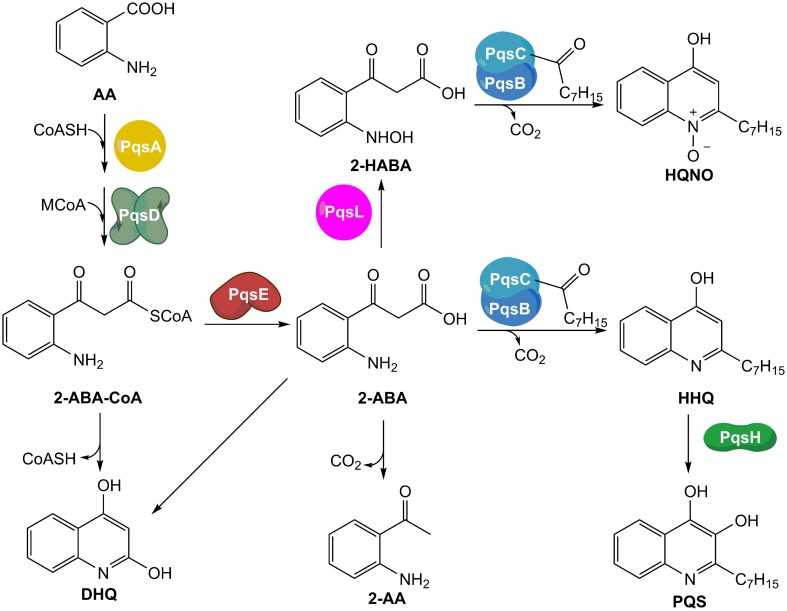
**Current model of the biosynthetic pathway of MvfR-related small molecules.** AA, anthranilic acid; CoASH, Coenzyme A; MCoA, malonyl-CoA; 2-ABA-CoA, 2′-aminobenzoylacetyl-CoA; 2-ABA, 2′-aminobenzoylacetate; DHQ, dihydroxyquinoline; 2-AA, 2′-aminoacetophenone; 2-HABA, 2′-hydroxylaminobenzoylacetate; HHQ, 4-hydroxy-2-heptylquinoline; HQNO, 4-hydroxy-2-heptylquinoline-N-oxide; PQS, *Pseudomonas* Quinolone Signal.

Briefly, the synthesis of HHQ and PQS starts with the conversion of anthranilic acid (AA) into its Coenzyme A (CoA) thioester derivative by the action of CoA-ligase PqsA. The activated molecule is then condensed with malonyl-CoA by PqsD leading to the formation of 2′-aminobenzoylacetyl-CoA (2-ABA-CoA), which is subsequently hydrolyzed by the thioesterase PqsE or TesB into 2′-aminobenzoylacetate (2-ABA) (Dulcey et al., 2013; Drees and Fetzner, 2015). This reactive intermediate is transformed into HHQ by the heterodimer PqsBC bearing an octanoyl chain (Dulcey et al., 2013). Finally, PqsH oxidizes HHQ into PQS (Schertzer et al., 2010) (**Figure 1**).

Furthermore, 2-ABA-CoA and 2-ABA are intermediates for the biosynthesis of other important metabolites. Actually, both compounds can cyclize leading to the formation of dihydroxyquinoline (DHQ), which has been shown to be fundamental in *P. aeruginosa* pathogenicity (Gruber et al., 2016), and in reducing the growth of epithelial cells (Zhang et al., 2008). Moreover, after decarboxylation, 2-ABA is converted into 2′-aminoacetophenone (2-AA), an important signal molecule that coordinates the transition from acute to chronic infection and the development of persister cells (Kesarwani et al., 2011; Que et al., 2013). In addition, 2-ABA could be converted into its hydroxylamine form by the oxidase PqsL and, then, transformed into 4-hydroxy-2-heptylquinoline-N-oxide (HQNO) by the complex octanoyl-PqsBC (Dulcey et al., 2013). HQNO is essential for biofilm formation because it favors extracellular DNA release by programmed cell lyses of the bacteria (Hazan et al., 2016).

Among the potential targets for blocking the *pqs* system, we herein discuss the transcriptional regulator MvfR and the biosynthetic enzyme PqsBC. So far, a number of MvfR antagonists and PqsBC inhibitors have been developed that efficiently reduced HHQ and PQS production in *P. aeruginosa* (Zender et al., 2013; Lu et al., 2014a; Starkey et al., 2014). The aim of this work was to gather detailed information about the effects of these QS Inhibitors (QSIs) on the production of MvfR-related small molecules including 2-AA, DHQ, HQNO, HHQ, and PQS. Furthermore, we monitored the expression of the *pqs* operon in a time-dependent manner upon treatment with the aforementioned QSIs.

Among the QS molecules, 2-AA was proven to be important in the development of *P. aeruginosa* persister cells (Que et al., 2013), which are individuals within the bacterial population characterized by having a reduced metabolism (Lewis, 2010). Due to their dormant state, antibiotic efficacy is severely impaired in this bacterial sub-group. Targeting persistence by blocking 2-AA production through QS inhibition was shown to be a promising strategy (Starkey et al., 2014). Consequently, an additional goal of the work was to quantify persister phenotype of *P. aeruginosa* after treatment with QSIs.

## Materials and Methods

### Chemicals and Growth Media

**FIGURE 2:**
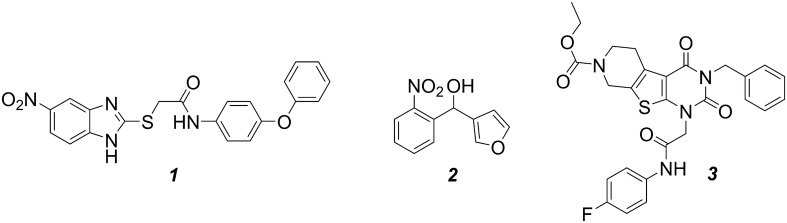
**Structures of the compounds evaluated in this work.** MvfR antagonist ***1***, PqsBC inhibitors ***2*** and ***3***.

The benzimidazole ***1*** and the nitrophenylmethanol ***2*** (**Figure 2**) were synthesized as described in literature (Rahme et al., 2012; Storz et al., 2014). *d_4_*-HHQ was synthesized following the procedure of HHQ using *d_5_*-aniline (Lu et al., 2012). The tricyclic ***3*** (**Figure 2**) and amitriptyline were purchased from ChemDiv (United States) and Alfa Aesar (Germany), respectively.

Water (Th. Geyer, Germany), acetonitrile (VWR, Germany) methanol (Sigma–Aldrich, United States) and formic acid (Fluka, United States) were LC-MS grade and used for HPLC-MS/MS experiments.

Yeast extract (Fluka, Germany), sodium chloride (VWR, Germany) and peptone from casein (Merck, Germany) were used for the preparation of Luria Bertani (LB) broth needed for performing the quantification experiments of *pqs*-related signal molecules. Ready-made mixture of LB broth (Fisher, United States) and Tryptic Soy Broth (TSB) 1% [*w/v*] (BD, United States) were used for pqsA-GFP_ASV_ and persister cells experiments.

### Bacterial Strains

*P. aeruginosa* PA14 and its isogenic transposon mutants *pqsA*, *pqsC*, *pqsH* and *pqsE* kindly provided by Susanne Häussler (Twincore, Hannover, Germany) were used in the experiments for the quantification of MvfR-related small molecules with and without QSIs. *P. aeruginosa* PA14, its isogenic *pqsBC* transposon mutant, and its *mvfR* single mutant were employed for performing persister cells assays. *P. aeruginosa* PA14 transformed with pAC37 carrying pqsA-GFP_ASV_ and gentamicin resistance cassette was used in *pqsA* expression experiments. The bacterial strains were maintained at -80°C in 25% [*v/v*] glycerol stocks.

### MvfR-Related Small Molecule Quantification

*Pseudomonas aeruginosa* strains were cultivated as previously described (Maurer et al., 2013). After letting PA14 strains grow for 6 h in LB, an aliquot of bacterial culture was centrifuged for 10 min at 4835 × *g* and 25°C using a Rotina 380R Centrifuge (Hettich, Germany). The supernatant was discarded, the pellet was resuspended in fresh LB, and the bacterial cells were spun down using the previous centrifugation settings. After repeating the wash step a second time, the OD_600_ of the washed cells was measured using a BioPhotometer plus Spectrophotometer (Eppendorf, Germany) in order to prepare a final bacterial suspension with OD_600_ = 0.02 in LB. Aliquots of 1.5 mL were added in each well of a 24-well Cellstar plate (Greiner Bio-One, Frickenhausen, Germany). Fifteen microliters of dimethyl sulfoxide (DMSO) or DMSO stock solutions of the target compound were added in each well. Triplicates of each condition were evaluated in every assay. The plates were incubated for 17 h at 37°C with 75% humidity and shaken at 200 rpm. The quantification of AA derivatives was performed following a previously described protocol (Lépine et al., 2003) with slight modifications. Seven hundred and fifty microliters of culture from each well were diluted with 750 μL of Internal Standard (IS) stock solution in acetonitrile. The IS employed in experiments with PA14 *wt* and *pqsC* mutant was *d_4_*-HHQ at a final concentration of 500 nM, while the experiments in PA14 *pqsH* mutant required amitriptyline as IS at a final concentration of 1 μM. After mixing the diluted cell culture for 5 min, the mixture was spun down for 15 min at 18,620 × *g* and 15°C using a Mikro 220R Centrifuge (Hettich, Germany). One milliliter of supernatant was transferred in glass vial and analyzed with the Accela HPLC system (Thermo Fisher Scientific, Germany) coupled to a triple quadrupole mass spectrometer TSQ Quantum Access Max (Thermo Fisher Scientific Germany) equipped with an HESI-II source. Separation was achieved by a Macherey-Nagel Nucleodur C_18_ Pyramid column (150 mm × 2 mm, 3 μm) heated at 40°C. The mobile phase consisted of 10:90 MeOH:H_2_O for 0.5 min, followed by a linear gradient of 1.5 min for reaching 100% MeOH, which was kept constant for 1 min. Then, the initial eluents composition was pumped for 2 min. The flow rate employed was 600 μL⋅min^-1^. A final concentration of 0.1% formic acid was present in the eluents. Compounds were ionized using heated electrospray ionization (hESI) in positive ion mode with the following parameters: spray voltage: 3500 V; vaporizer temperature: 370°C; sheath gas pressure (nitrogen): 35 units; auxiliary gas pressure (nitrogen): 30 units; skimmer offset voltage: 0 V; capillary temperature: 270°C. Selected reaction monitoring was used for detecting DHQ (161.971→115.979, collision energy: 28 V, tube lens: 95 V), 2-AA (136.016→91.048, collision energy: 24 V, tube lens: 68 V), HHQ (244.050→158.944; collision energy: 31 V; tube lens: 100 V), HQNO (260.036→158.908; collision energy: 28 V; tube lens: 110 V), PQS (260.048→174.927; collision energy: 30 V; tube lens: 110 V), *d_4_*-HHQ (248.081→162.965; collision energy: 32 V; tube lens: 100 V) and amitriptyline (278.061→232.932; collision energy: 16 V; tube lens: 90 V) employing a scan width of 0.010 *m/z*, a scan time of 0.100 s, and a peak width of 0.70. Calibration curves were prepared following the same protocol and using PA14 *pqsA* mutant as matrix without compounds and spiked with known concentrations of analytes and IS after the overnight growth. The assays were repeated at least three times.

### *pqsA* Expression Assay

The assays were performed following the protocol previously published (Kesarwani et al., 2011) with few modifications. PA14 *wt* cells transformed with pAC37 were grown overnight in LB with 60 μg/mL of gentamicin, then an aliquot of bacterial culture was centrifuged for 5 min at 8000*g* at 25°C using a 5810R Centrifuge (Eppendorf, United States). The supernatant was discarded, the pellet was resuspended in fresh LB with the same antibiotic, and the bacterial cells were spun down using the previous centrifugation settings. After repeating the wash step a second time, the OD_600nm_ of the washed cells was measured using a Spectronic Unicam Genesys 10 UV spectrophotometer (Thermo Fisher, United States) in order to prepare a final bacterial suspension with OD_600nm_ = 0.02 in LB with 60 μg/mL of gentamicin. Hundred microliters of the prepared culture was poured in each well of a 96-well plate (Corning Inc., Corning, NY, United States) and the compounds were added in triplicates. The final concentration of DMSO was 1% v/v. The plates were incubated at 37°C under static condition in Infinite F200 plate reader (Tecan Group Ltd, Männedorf, Switzerland) monitoring GFP fluorescence (λ_ex_ = 485 nm; λ_em_ = 535 nm) and OD_600nm_ after a short shaking every 15 min. The assays were repeated at least three times.

### Persister Cells Assay

The effects of QSIs on persistence were evaluated following the published protocol (Starkey et al., 2014) with some modifications. After streaking the bacteria on LB agar and overnight incubation at 37°C, one colony was dispersed in 5 mL of LB and the bacteria were grown at 37°C up to OD_600nm_ = 0.5. Thirty microliters of the culture were transferred into glass tubes with 5 mL of TSB 1% [*w/v*] and incubated overnight at 37°C under shaking condition. Then, an aliquot of *P. aeruginosa* culture was centrifuged for 5 min at 8,000 × *g* and 25°C using a 5810R Centrifuge (Eppendorf, United States). The supernatant was discarded, the pellet was resuspended in fresh LB, and the bacterial cells were spun down using the previous centrifugation settings. After repeating the wash step a second time, the OD_600nm_ of the washed cells was measured using a Spectronic Unicam Genesys 10 UV spectrophotometer (Thermo Fisher, United States) in order to prepare a final bacterial suspension with OD_600nm_ = 0.02 in 5 mL of TSB 1% [*w/v*] in each glass tube. The target compounds were added in the tubes and the final concentration of DMSO was 0.5% [*v/v*]. The bacterial suspension was incubated at 37°C under shaking condition for 4 h. An aliquot of 100 μL of culture from each tube was used for dilution plating on LB agar plates and colony forming units (CFU) quantification (normalizers). Fifty microliters of meropenem 1 mg/mL were added in each tube and the cultures were incubated at 37°C for 24 h under shaking condition. Aliquots of 600 μL of bacterial suspension were utilized for dilution plating on LB agar plates and CFU quantification (persisters). The survival fractions were calculated as the ratio of normalizers over persisters. Triplicates per each condition were employed in each assay and the experiments were repeated at least three times.

## Results and Discussion

The QSIs evaluated in this study were the most potent developed MvfR antagonist ***1*** (Starkey et al., 2014), and the two PqsBC inhibitors ***2*** (Starkey et al., 2014) and ***3*** (this study) shown in **Figure 2**. As previously published, these compounds were able to inhibit dose-dependently the production of the signal molecules HHQ and PQS in PA14 *pqsH* mutant and *wt*, respectively. However, a complete profiling of all MvfR-related small molecules was not performed using different concentrations of QSIs. To ensure a convenient analytic procedure, we developed an “all-in-one” method for the simultaneous evaluation of these bacterial metabolites. Following the protocol by Lépine et al. (2003) with some optimizations, a single assay for quantification of the relevant *pqs*-related small molecules was established allowing medium throughput, easy sample processing, low material consumption, without relevant interference between analytes.

### Effects of MvfR Antagonists on *pqs*-Related Small Molecules

**FIGURE 3:**
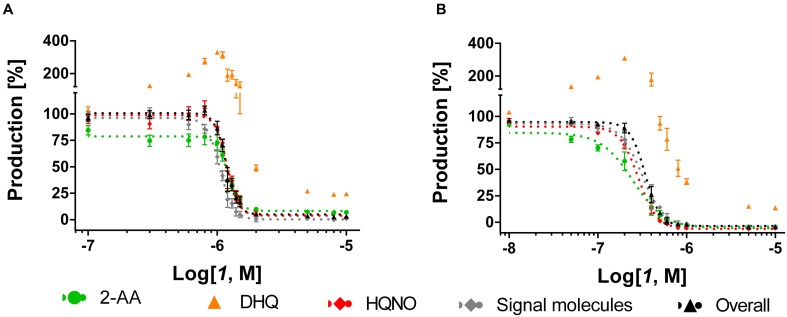
**Dose-response curves of MvfR antagonist ***1*** acting on MvfR-related small molecules production in PA14 *wt*** **(A)** and PA14 *pqsH* mutant **(B)**. 2-AA: green. DHQ: orange. Signal molecules: gray. HQNO: red. Sum of all anthranilic acid derivatives: black. The “*x*” axes indicate the logarithm of the concentration of the antagonists in molar units (M). The error bars indicate Standard Error of the Mean.

As previously shown (Starkey et al., 2014), the MvfR antagonist ***1*** was able to reduce dose-dependently the production of the alkyl-quinolones (AQs) and 2-AA in PA14 *wt* with an IC_50_ (inhibitor concentration causing half maximum inhibition) of 1.1 μM (**Table 1**; **Figure 3A**). Interestingly, the sigmoidal curves of each of these metabolites were very steep with a Hill coefficient over 1 giving the idea of a possible exponential effect of the MvfR natural ligands on the *pqs* regulon expression. Moreover, while DHQ production was inhibited at high concentrations, its biosynthesis was enhanced up to 330% after incubation of *P. aeruginosa* with ***1*** at a concentration close to the compound’ IC_50_. The basal levels of DHQ were reached at lower doses of the QSI. Nevertheless, ***1*** was able to reduce dose-dependently the overall biosynthesis of these metabolites with an IC_50_ of 1.2 μM.

**Table 1:** Effects of the MvfR antagonist ***1*** on production of MvfR-related small molecules in PA14 *wt* and PA14 *pqsH* mutant.

Strains	2-AA IC_50_ [μM]	Maximal DHQ production [%]	HQNO IC_50_ [μM]	Signal molecules^a^ IC_50_ [μM]	Overall IC_50_ [μM]
**PA14 *wt***	1.3 (1.1-1.2)^b^	330 ± 12^c^ @ 1 μM	1.2 (1.1-1.3)^b^	1.1 (1.0-1.2)^b^	1.2 (1.1-1.3)^b^
**PA14 *pqsH* mutant**	0.19 (0.16-0.22)^b^	304 ± 2^c^ @ 0.2 μM	0.27 (0.25-0.30)^b^	0.27 (0.25-0.30)^b^	0.32 (0.30-0.34)^b^


*^a^In PA14 *wt* referred to sum of HHQ and PQS concentrations; in PA14 *pqsH* mutant referred to HHQ concentration. ^b^95% Confidence Intervals. ^c^Standard Error of the Mean intervals.*

For confirming this characteristic profile, the compound was also evaluated in PA14 *pqsH* mutant strain, which does not convert HHQ into PQS. Notably, in a clinical setting, it has been observed that *P. aeruginosa* tends to produce much more HHQ than the hydroxylated analog (Que et al., 2011). Our experiments with the PQS-deficient *pqsH* mutant revealed that the QSIs showed similar profiles on the other *pqs*-related molecules production compared to PA14 *wt*. Indeed, ***1*** efficiently inhibited AQs and 2-AA production displaying an IC_50_ of circa 0.30 μM, and very steep inhibitory curves as in PA14 *wt* (**Table 1**; **Figure 3B**). Likewise, they enhanced DHQ formation up to 300% at AQs IC_50_ concentration. Considering that PQS is more active than HHQ in inducing *pqs* expression (Xiao et al., 2006), it is not surprising that the MvfR antagonists were more potent in repressing the biosynthesis of *pqs*-related signal molecules in PA14 *pqsH* mutant than in *wt*.

The characteristic profiles in *wt* and *pqsH* mutant of this class of QSIs, such as the steep inhibitory dose-dependent curves and the overproduction of DHQ close to the IC_50_ for AQ inhibition, suggest the *pqs* autoloop as a reason. Actually, PQS and HHQ have high activity toward MvfR in the nanomolar range (Xiao et al., 2006; Lu et al., 2014b) and the actual QS signal is amplified through expression of enzymes, which produce again a multitude of additional MvfR natural ligands. Antagonizing the transcriptional regulator would thus have a higher-order effect on the downstream products resulting from pseudo-cooperative effects. Each decrease in signal molecule synthesis achieved by MvfR antagonism would have an additional impact on MvfR deactivation due to less competing autoinducers. In the concentration range close to the antagonist IC_50_, it would be plausible that the biosynthetic pathway cannot convert the major amount of AA into HHQ and PQS maybe because of a slow kinetic step in the biosynthesis. Considering the low affinity of 2-ABA toward PqsBC (Drees et al., 2016), it is feasible to claim that the slow step is the condensation and cyclization reaction performed by PqsBC. This would lead to an accumulation of the reactive intermediate 2-ABA that quickly cyclizes into DHQ.

**FIGURE 4:**
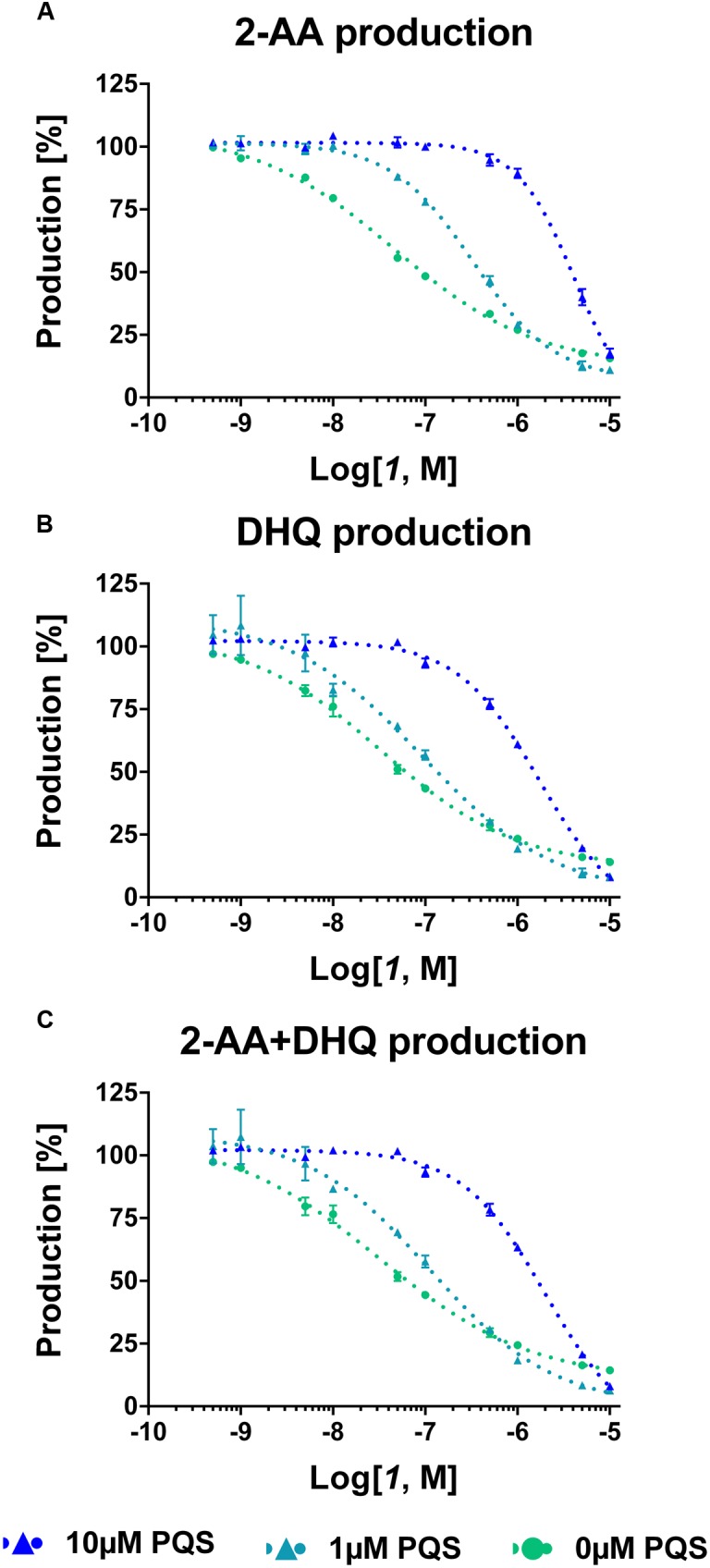
**Dose-response curves of MvfR antagonist ***1*** on 2-AA** **(A)**, DHQ **(B)** and overall **(C)** production in PA14 *pqsC* mutant with and without external addition of PQS. The “*x*” axes indicate the logarithm of the concentration of the antagonists in molar units (M). The error bars indicate Standard Error of the Mean.

For confirming these hypotheses, the compound was evaluated in PA14 *pqsC* mutant, which synthesizes only 2-AA and DHQ, in an experimental setup with and without exogenous addition of the signal molecule PQS. Since these bacteria do not produce any MvfR natural ligands, the *pqs* autoloop is consequently absent and it was possible to control its expression with external administration of PQS. Exogenous addition of the quinolone at 1 and 10 μM reduced the potency of the QSIs on MvfR-related small molecules synthesis of circa one and two orders of magnitude, respectively, and increased the steepness of the inhibitory curves (**Table 2**; **Figure 4**). Actually, the IC_50_ of ***1*** on 2-AA production worsened from 50 nM (without PQS) over 0.3 μM (with 1 μM PQS) to 4.2 μM (10 μM PQS). In addition, the concentration of compound needed to reduce DHQ production to 50% shifted from 30 nM (without PQS) over 90 nM (1 μM PQS) to 1.7 μM (10 μM PQS). Consequently, the overall effect of the QSI on the production of *pqs*-related molecules within the PqsADE biosynthetic pipeline present in the *pqsC* mutant was also affected by PQS administration. Hence, an increase of IC_50s_ from 30 nM (no PQS) over 0.11 μM (1 μM PQS) to 2.0 μM (10 μM PQS) was observed.

**Table 2:** Effects of MvfR antagonist ***1*** on 2-AA and DHQ production in PA14 *pqsC* mutant with and without external addition of PQS.

Compounds	Exogenous PQS [μM]	2-AA IC_50_ [μM]	DHQ IC_50_ [μM]	2-AA + DHQ IC_50_ [μM]
***1***	0	0.05 (0.04-0.06)	0.03 (0.03-0.04)	0.03 (0.02-0.04)
	1	0.33 (0.28-0.38)	0.09 (0.05-0.18)	0.11 (0.07-0.19)
	10	4.2 (3.0-5.8)	1.7 (1.3-2.3)	2.0 (1.4-2.8)


*The data are reported with 95% Confidence Interval.*

These findings confirmed that the presence of PQS in the bacterial culture plays an important role in controlling the biosynthesis of *pqs*-related molecules. Indeed, when the natural ligand is present, the efficiency of the compounds was strongly reduced as the shifts in IC_50_ confirmed. In addition, the increased steepness of the inhibitory curves after addition of PQS to the PA14 *pqsC* mutant culture displayed that, in case of a small reduction in concentration of QSIs, the MvfR-related compounds were quickly restored to the basal production level.

**FIGURE 5:**
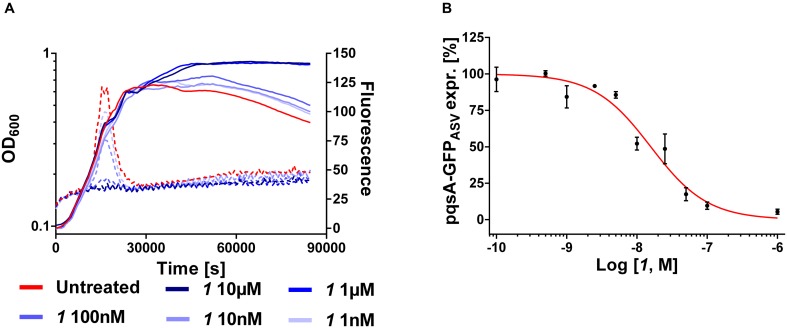
**Expression of pqsA-GFP_ASV_ (dotted lines) and growth curves (solid lines) of *Pseudomonas aeruginosa* treated with ***1*(A)**.** IC_50_ curve of ***1*** **(B)**. The “*x*” axes indicate the logarithm of the concentration of the antagonists in molar units (M). The error bars indicate Standard Error of the Mean.

Furthermore, the expression levels of *pqsA* were monitored under MvfR antagonist treatment using the PA14 *wt* transformed with the plasmid carrying the construct *pqsA*-GFP_ASV_ (Yang et al., 2007). As previously reported (Kesarwani et al., 2011), the kinetic studies showed that the expression of the *pqsA* promoter present in the *pqsA*-GFP_ASV_ plasmid occurred at the exponential phase of bacterial growth as it was analyzed for the genomic *pqs* operon (Déziel et al., 2004). As formerly evaluated (Starkey et al., 2014), ***1*** efficiently inhibited the promoter expression displaying an IC_50_ of 16 nM (**Figure 5**).

Combining these findings with the results in PA14 *wt* and *pqsH* mutant indirectly confirmed that the action of autoinducers HHQ and PQS within this positive feedback loop is the explanation for the pronounced steepness of the sigmoidal dose-response curves for 2-AA, HHQ, PQS, and HQNO levels. The fact that down-regulation of the *pqs* operon results in an initial increase in DHQ, while the other investigated components show the expected sigmoidal dose-response curves, suggests that either 2-ABA-CoA or 2-ABA accumulate in this scenario, which can be spontaneously degraded to the shunt product. From published studies on the whole PQS biosynthesis cassette, it can be assumed that the reactions mediated by the PqsAD-cascade proceed quickly enough also at lowered enzyme concentrations to enable sufficient 2-ABA-CoA production. These initial steps should be more dependent on the cellular availability of the substrates AA and malonyl-CoA. The hydrolysis catalyzed by PqsE can also be performed by housekeeping thioesterases like TesB. But, an analysis of a *pqsE* transposon mutant by Drees and Fetzner (2015) regarding the profile of PQS-related molecules showed increased DHQ levels. We corroborated and extended the data on this strain with the established *pqs*-related small molecule quantification method (Supplementary Figure S1). As expected for this strain, 2-AA levels are reduced while DHQ concentration is dramatically increased hinting at accumulation of the reactive intermediate 2-ABA-CoA. Finally, insufficient action of PqsBC and, thus, accumulation of 2-ABA should lead to both increased DHQ and 2-AA levels.

Hence, the gathered information suggests that through the MvfR-antagonist-induced down-regulation of the *pqs* operon an initial accumulation of 2-ABA-CoA rather than 2-ABA takes place (as in the *pqsE* mutant). Interestingly, it has been shown that the condensation reaction mediated by PqsBC should be the rate-limiting step in this biosynthetic cascade (Drees and Fetzner, 2015). Thus, we consider it rather surprising that we observe a metabolite profile, that hints more toward the role of 2-ABA-CoA upon partial inactivation of the *pqs* operon.

Nevertheless, at very low levels of MvfR activity, DHQ is finally reduced as the diminished PqsAD cascade ceases to operate efficiently resulting in the observed metabolite profiles.

**FIGURE 6:**
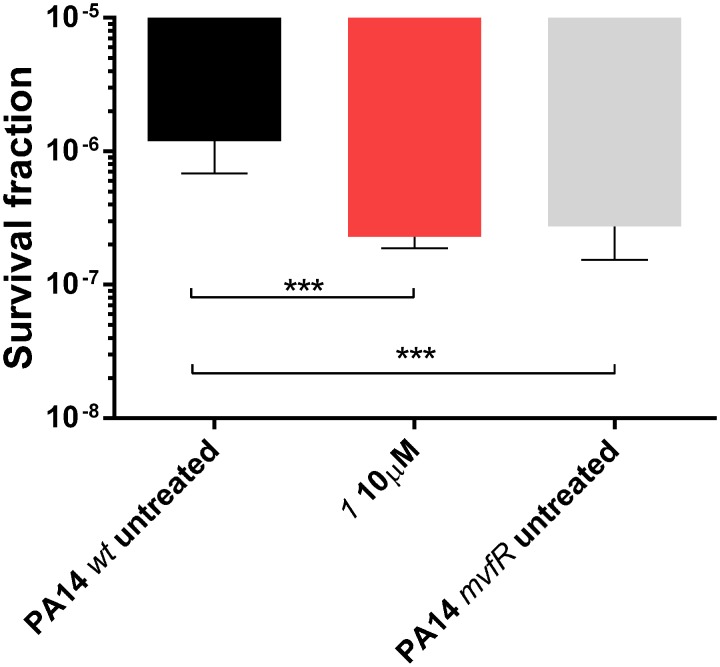
**Persister cells survival of PA14 *wt* with and without MvfR antagonist ***1*** and PA14 *mvfR* mutant after the treatment with 10 μg/mL of meropenem for 24 h.** The error bars indicate 95% Confidence Interval of the geometric mean. Statistical analysis performed with non-parametric one-way ANOVA (α = 0.05; ^∗∗∗∗^*p* < 0.0001; ^∗∗∗^*p* < 0.003; ^∗^*p* < 0.05).

Among the *pqs*-related molecules produced by this pathogen, 2-AA was shown to be a key factor for switching the infection from acute to chronic state favoring the formation of persister cells (Kesarwani et al., 2011; Que et al., 2013). Actually, high levels of this QS compound enhanced the genesis of antibiotic tolerant cells within the population reducing the killing activity of antibiotics. Considering the high efficiency of the MvfR antagonists in reducing 2-AA (Starkey et al., 2014), these QSIs were evaluated in their capability to modify the survival fraction of the pathogen after treatment with meropenem. A dose of 10 μM of ***1*** reduced significantly persisters development in PA14 *wt* from 1.2 × 10^-6^ of the untreated control to 2.3 × 10^-7^ reaching the same levels of the 2-AA non-producing strain PA14 *mvfR* mutant, that is 2.7 × 10^-7^ (**Figure 6**) as previously demonstrated (Starkey et al., 2014). These findings corroborated the importance of suppressing the biosynthesis of this carbonyl compound via blockage of the transcriptional regulator for achieving a more efficient antibiotic therapy against *P. aeruginosa* infections.

### Effects of PqsBC Inhibitors on *mvfR*-Related Small Molecules

**FIGURE 7:**
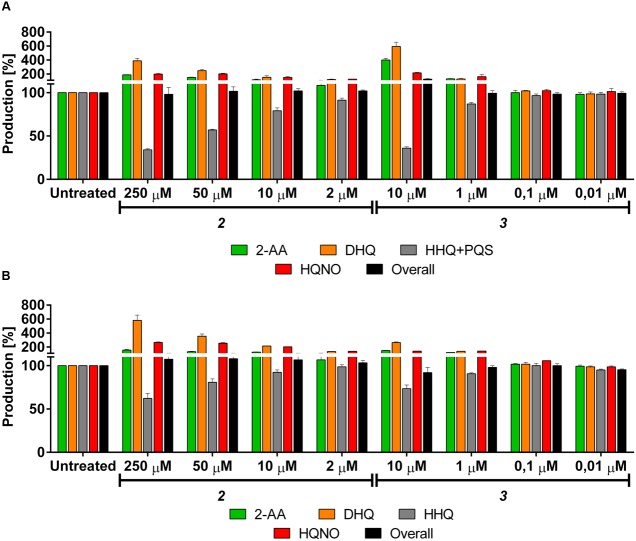
**Effects of PqsBC inhibitors on *pqs* related signal molecules production in PA14 strains.** **(A)** ***2*** and ***3*** in PA14 *wt*. 2-AA: green. DHQ: orange. PQS + HHQ: gray. HQNO: red. Sum of all anthranilic acid derivatives: black. **(B)** ***2*** and ***3*** in PA14 *pqsH* mutant. 2-AA: green. DHQ: orange. HHQ: gray. HQNO: red. Sum of all anthranilic acid derivatives: black. The error bars indicate Standard Error of the Mean. Statistical analysis performed with one-way ANOVA (α = 0.05).

The PqsBC inhibitors ***2*** and ***3*** reduced the production of the MvfR natural ligands in PA14 *wt* down to 34 and 35%, respectively, at their highest concentration (**Table 3**; **Figure 7A**). As expected (*vide supra*), the levels of 2-AA and DHQ strongly increased after the treatment with such inhibitors up to 188 and 389% after incubation with 250 μM of ***2*** and 415 and 654% with 10 μM of ***3***. Surprisingly, the synthesis of HQNO was also enhanced up to two times compared to the untreated bacteria. Interestingly, the overall production of the AQ signal molecules was not significantly affected. Reducing the concentration of these QSIs led to a reduced inhibitory activity on HHQ and PQS production and a relapse of 2-AA, DHQ and HQNO to the respective basal levels.

**Table 3:** Production of *pqs* signal molecules in PA14 *wt* after the treatment with PqsBC inhibitors.

Compounds	Concentration [μM]	2-AA [%]	DHQ [%]	HQNO [%]	HHQ + PQS [%]	Overall [%]
***2***	250	188 ± 4	389 ± 33	198 ± 12	34 ± 1	98 ± 8
	50	152 ± 4	249 ± 15	201 ± 10	57 ± 1	101 ± 5
	10	111 ± 8	141 ± 31	148 ± 19	81 ± 6	100 ± 3
	2	107 ± 2	115 ± 7	128 ± 1	93 ± 4	102 ± 2
***3***	10	415 ± 39	654 ± 49	218 ± 16	35 ± 3	136 ± 5
	1	134 ± 7	131 ± 12	181 ± 39	86 ± 2	100 ± 5
	0.1	99 ± 4	102 ± 1	103 ± 2	96 ± 3	98 ± 3
	0.01	99 ± 4	100 ± 4	97 ± 2	103 ± 5	99 ± 3


*100% is the level of metabolite produced in the untreated PA14 *wt*. The data are reported with Standard Error of the Mean intervals.*

For confirming this characteristic profile, the compounds were also evaluated in PA14 *pqsH* mutant and similar results were obtained compared to the isogenic *wt* (**Table 4**; **Figure 7B**). Here, ***2*** and ***3*** reduced at their highest dosage the formation of HHQ down to 62 and 73%, respectively. Moreover, the production of 2-AA, DHQ, and HQNO was increased by 157, 581, and 265% after treatment with 250 μM of ***2*** and 150, 264, and 141% after incubation with 10 μM of ***3*** compared to the untreated control. In addition, as in PA14 *wt*, the overall amount of the MvfR-related compounds was not affected by the addition of these QSIs.

**Table 4:** Production of *pqs* signal molecules in PA14 *pqsH* mutant after the treatment with PqsBC inhibitors.

Compounds	Concentration [μM]	2-AA [%]	DHQ [%]	HQNO [%]	HHQ [%]	Overall [%]
***2***	250	157 ± 8	581 ± 76	265 ± 8	62 ± 6	107 ± 4
	50	134 ± 5	355 ± 31	255 ± 10	81 ± 4	108 ± 3
	10	126 ± 6	215 ± 3	199 ± 2	91 ± 4	104 ± 4
	2	102 ± 2	133 ± 2	138 ± 1	97 ± 2	101 ± 2
***3***	10	150 ± 3	264 ± 11	141 ± 1	73 ± 4	92 ± 6
	1	121 ± 2	139 ± 3	143 ± 2	91 ± 1	98 ± 2
	0.1	102 ± 1	102 ± 2	106 ± 1	100 ± 3	100 ± 2
	0.01	99 ± 1	99 ± 1	99 ± 1	95 ± 1	95 ± 1


*100% is the level of metabolite produced in the untreated PA14 *pqsH* mutant. The data are reported with Standard Error of the Mean intervals.*

**FIGURE 8:**
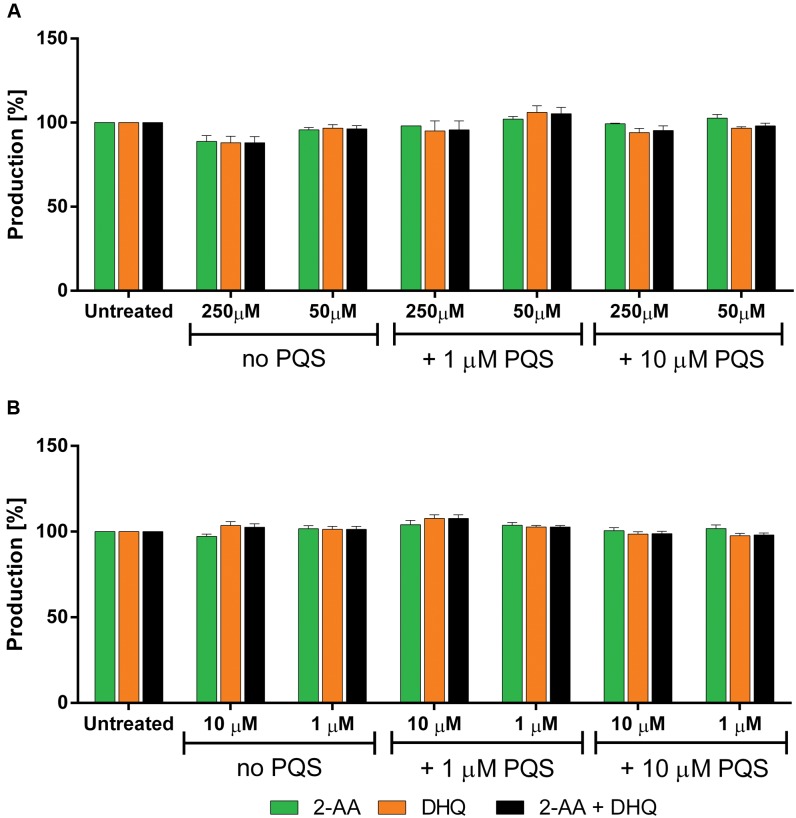
**Effects of PqsBC inhibitors on 2-AA and DHQ production in PA14 *pqsC* mutant.** **(A)** ***2*** and **(B)** ***3***. 2-AA: green. DHQ: orange. Sum of all anthranilic acid derivatives: black. The error bars indicate Standard Error of the Mean. Statistical analysis performed with one-way ANOVA (α = 0.05).

Additionally, ***2*** and ***3*** were examined in PA14 *pqsC* mutant for confirming their target selectivity. Both compounds turned out to be inactive in reducing 2-AA and DHQ production independently from the concentration of PQS added into the culture (**Figure 8**) supporting the *in vitro* characterization of the two inhibitors (unpublished results; Starkey et al., 2014).

**FIGURE 9:**
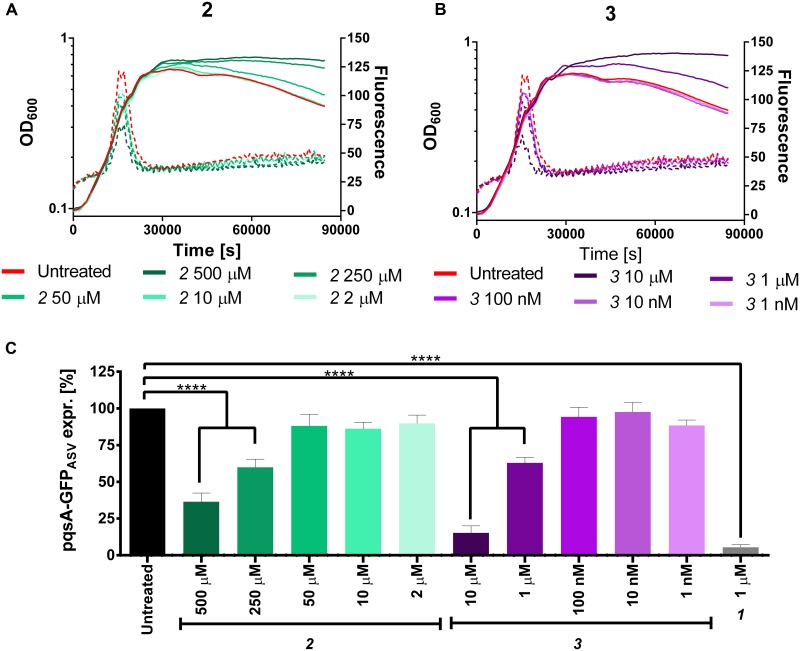
**Expression of pqsA-GFP_ASV_ (dotted lines) and growth curves (solid lines) of *Pseudomonas aeruginosa* treated with ***2*(A)**** and *3* **(B)**. **(C)** Percentage of pqsA-GFP_ASV_ expression after PqsBC inhibitors addition. The error bars indicate Standard Error of the Mean. Statistical analysis performed with one-way ANOVA (α = 0.05; ^∗∗∗∗^*p* < 0.0001).

Considering their efficiency in reducing the production of the MvfR natural ligands in PA14 *wt* and *pqsH* mutant, these inhibitors were analyzed in the pqsA-GFP_ASV_ construct for monitoring their potential ability for reducing the expression of the *pqs* operon. Actually, ***2*** and ***3*** reduced the operon transcription down to 36 and 15%, respectively, at their highest dosage (**Figure 9**). However, it is not clear in this setting why no autolysis is observed despite the presence of HQNO, which is responsible for the programmed cell autolysis observed in liquid cultures of *P. aeruginosa* (Hazan et al., 2016).

Taking into consideration the obtained results, the effects of the PqsBC inhibitors on the MvfR natural ligands, 2-AA, and DHQ production fitted to the expected behavior of blocking the hetero-dimer PqsBC. Actually, its inhibition would lead to a reduced conversion of the reactive 2-ABA into HHQ with consequently reduced expression of the *pqs* operon. The excess of this intermediate would be consequently transformed more into the stable molecules 2-AA and DHQ, conversions that do not require PqsBC. Notably, as previously reported, PqsBC inhibitor ***3*** efficiently reduced HQNO, HHQ and PQS production at a concentration of around 90 μM (Starkey et al., 2014). Hence, full PqsBC inhibition occurs at concentrations higher than the ones used in the present study, which hints to a similar concentration dependency as observed for DHQ levels upon application of MvfR antagonists. According to the current model of HQNO synthesis, 2-ABA is *N*-oxidized by the oxidase PqsL into its hydroxylamine form, 2-HABA. We assume that this moiety of the intermediate should be a more reactive nucleophile (Ningst et al., 2012) than the amine of 2-ABA or a better substrate and that, consequently, the reaction of condensation and cyclization performed by the hetero-dimer proceeds faster. Moreover, it is plausible to expect that the enzyme complex has a different affinity toward the amine and the hydroxylamine intermediates and, due to this fact, the inhibitors would have also different activities in blocking both reactions. Based on the obtained results, this hypothesis would reasonably explain the overproduction of HQNO after incubation of PA14 with PqsBC inhibitors. But, we do not exclude that an additional, yet unknown bypass reaction toward HQNO might cause this unexpected observation. In addition, the analysis of the overall amount of the *pqs*-related small molecules revealed that these QSIs modify only the ratio of the QS compounds without affecting the total content.

**FIGURE 10:**
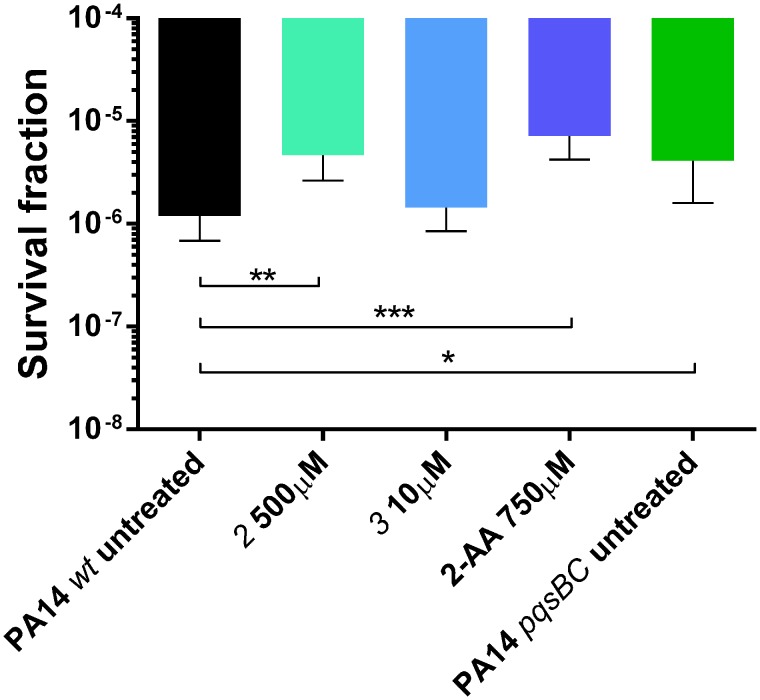
**Persister cells survival of PA14 *wt* with and without PqsBC inhibitors ***2*** and ***3***, or 2-AA and PA14 *pqsBC* mutant after the treatment with 10 μg/mL of meropenem for 24 h.** The error bars indicate 95% Confidence Interval of the geometric mean. Statistical analysis performed with non-parametric one-way ANOVA (α = 0.05; ^∗∗∗^*p* < 0.003; ^∗∗^*p* < 0.01; ^∗^*p* < 0.05).

As described above, QS profiles observed for PqsBC inhibitors were different from those determined for the MvfR antagonist. In particular, the inductive effects on 2-AA production drew our attention. Hence, we examined ***2*** and ***3*** regarding their ability to affect the development persister cells (as reported in Starkey et al., 2014). We found that only ***2*** significantly enhanced the persistence phenotype of PA14 *wt* increasing the survival fraction from 1.2 × 10^-6^ (untreated) up to 4.6 × 10^-6^ cells. This corresponds to the levels of PA14 *wt* treated with 2-AA as well as the untreated PA14 *pqsBC* mutant showing persister rates of 7.2 × 10^-6^ and 4.1 × 10^-6^, respectively (**Figure 10**). These findings confirmed that targeting PqsBC led to survival of the bacteria in the presence of antibiotic. This might ultimately result in a reduced efficiency of antibiotic therapy.

## Conclusion

The profiling of *P. aeruginosa* MvfR antagonist and PqsBC inhibitors emphasized the importance in selecting the target for the development of new anti-infectives, and confirmed that MvfR is an excellent target for a global AQ and 2-AA inhibition. Actually, antagonizing the transcriptional regulator led to an efficient inhibition of the *pqs* operon expression and, consequently, of the *mvfR*-small molecules production. As it was found an overproduction of the toxic DHQ at AQs IC_50_ doses, the current study highlighted that an efficient therapy would be obtained only choosing a concentration of QSI capable to fully suppress the MvfR activity and, in the end, the biosynthetic pathway. The PqsBC inhibitors showed to be less efficient in reducing the MvfR natural ligands synthesis and, moreover, lead to an increased production of 2-AA and DHQ. Actually, they mainly affected the distribution of QS molecules generated within a bacterial population without modifying the overall production. The importance of reducing 2-AA production through MvfR antagonism is critical in preventing the formation of antibiotic tolerant persister cells. In case of PqsBC inhibitors, a combination with other QSIs (e.g., PqsA, PqsD, or MvfR) might still be a valid route toward the use of a novel anti-infective approach.

## Author Contributions

GA: Performed experiments and wrote the paper. CM and JE: Supervised LC-MS experiments and wrote the paper. DM and LR: Supervised cellular experiments. RH and ME: Supervised experiments and wrote the paper.

## Conflict of Interest Statement

The authors declare that the research was conducted in the absence of any commercial or financial relationships that could be construed as a potential conflict of interest.
