# Supplementary material for: In-depth Profiling of MvfR-Regulated Small Molecules in Pseudomonas aeruginosa after Quorum Sensing Inhibitor Treatment
Source: Front Microbiol. 2017 May 24;8:924. doi: 10.3389/fmicb.2017.00924 (PMC5442231; doi:10.3389/fmicb.2017.00924)
Supplement: Supplementary file 1 [file Data_Sheet_1.docx]

**In-depth Profiling of MvfR-regulated small molecules in *Pseudomonas aeruginosa* after Quorum Sensing Inhibitor treatment**

**Giuseppe Allegretta^1^, Christine K. Maurer^1^, Jens Eberhard^1^, Damien Maura^2,3,4^, Rolf W. Hartmann^1,5*^, Laurence Rahme^2,3,4*^, Martin Empting^1*^**

^1^Department of Drug Design and Optimization, Helmholtz Institute for Pharmaceutical Research Saarland, Saarbrücken, Germany

^2^Department of Surgery and Department of Microbiology and Immunobiology, Harvard Medical School, Boston, MA, USA

^3^Department of Surgery, Center for Surgery, Innovation and Bioengineering, Massachusetts General Hospital, Boston, MA, USA

^4^Shriners Hospitals for Children Boston, Boston, MA, USA

^5^Pharmaceutical and Medicinal Chemistry, Saarland University, Saarbrücken, Germany

***Supporting information***





**Figure S1** Relative production of 2-AA, DHQ, HQNO, HHQ + PQS and overall amount of AQs in PA14 *pqsE* mutant compared to PA14 *wt*. The error bars indicate Standard Error of the Mean.
